# Languages Support Efficient Communication about the Environment: Words for Snow Revisited

**DOI:** 10.1371/journal.pone.0151138

**Published:** 2016-04-13

**Authors:** Terry Regier, Alexandra Carstensen, Charles Kemp

**Affiliations:** 1 Department of Linguistics, Cognitive Science Program, University of California, Berkeley, CA 94720, United States of America; 2 Department of Psychology, University of California, Berkeley, CA 94720, United States of America; 3 Department of Psychology, Carnegie Mellon University, Pittsburgh, PA 15213, United States of America; Plymouth University, UNITED KINGDOM

## Abstract

The claim that Eskimo languages have words for different types of snow is well-known among the public, but has been greatly exaggerated through popularization and is therefore viewed with skepticism by many scholars of language. Despite the prominence of this claim, to our knowledge the line of reasoning behind it has not been tested broadly across languages. Here, we note that this reasoning is a special case of the more general view that language is shaped by the need for efficient communication, and we empirically test a variant of it against multiple sources of data, including library reference works, Twitter, and large digital collections of linguistic and meteorological data. Consistent with the hypothesis of efficient communication, we find that languages that use the same linguistic form for snow and ice tend to be spoken in warmer climates, and that this association appears to be mediated by lower communicative need to talk about snow and ice. Our results confirm that variation in semantic categories across languages may be traceable in part to local communicative needs. They suggest moreover that despite its awkward history, the topic of “words for snow” may play a useful role as an accessible instance of the principle that language supports efficient communication.

## Introduction

Franz Boas observed that certain Eskimo languages have unrelated forms for subtypes of snow (e.g. *aput*: snow on the ground, *qana*: falling snow), and thus subdivide the notion of snow more finely than English does [1]. He suggested that such cross-language variation in the grouping of ideas into named categories “must to a certain extent depend upon the chief interests of a people” [1]. Boas’ Eskimo example was repeated by Whorf [2], and was subsequently exaggerated through popularization, leading to grossly inflated claims about the number of words for snow in Eskimo languages. Through this exaggeration and resulting critique [3, 4], the snow example has acquired an air of unseriousness, and it tends to be avoided by many scholars. However, recent work has suggested some empirical support for the original claim prior to its distortion [5], motivating a broader re-examination across languages, and greater theoretical attention.

Although the snow example has been used to advance multiple and sometimes contradictory theoretical stances, we take the original motivation behind Boas’ example to concern the adaptation of languages to their physical environments, as summarized by the following causal chain:

$$\begin{array}{c} \text{Environment}\to \text{Communicative need}\to \text{Category system} \end{array}$$

That is, local physical environment (abundant snow in various forms) shapes local cultural communicative needs (“the chief interests of a people”, including the need to communicate precisely and informatively about snow), which in turn shape the category system of a language (narrow and precise semantic categories for subtypes of snow). Related reasoning underlies other proposed examples of local environmental influences on semantic categories, concerning names for visual lightness [6], body parts [7], and topographical features of the physical environment itself [8], as well as other aspects of language such as linguistic tone [9] and the ratio of sonorant to obstruent segments [10].

This reasoning is a special case of the more general hypothesis that language is shaped by the functional need for efficient communication—that is, communication that is informative and precise, yet requires minimal effort. Many aspects of language yield evidence consistent with this hypothesis, including word frequency distributions [11, 12], word length [13], syllable duration [14], compositional structure [15], syntactic structures and processing [16–18], the learning of case marking [19], and—of direct relevance to Boas’ snow example—systems of semantic categories across languages [20, 21]. From the perspective of efficient communication, a system of fine-grained categories is both more informative than a single broad category, and more complex, requiring more effort to store and process. The added complexity of a fine-grained system may be worth the investment if the gain in informativeness is compounded by frequent use of the fine-grained categories. This reasoning predicts that semantically fine-grained categories will tend to appear in frequently referenced parts of semantic space, as argued by Greenberg [22] and confirmed in a recent large-scale study of kinship systems across languages [20]. Here, we pursue the idea that local environment may shape frequency of reference to particular items, and that category systems may vary accordingly, as predicted by the hypothesis of efficient communication.

The debate touched off by the Eskimo snow example has continued to focus primarily on Eskimo/Inuit languages, but evaluating the full causal chain above in a convincing manner would require data from many languages. A useful starting point for such a cross-language study is provided by an existing observation. After echoing Boas’ proposal of a connection between colder temperature and semantic subdivision of the concept of snow, Whorf [2] extended the same principle in the opposite direction, arguing for a link between warmer temperature and the use of a single semantically broad morpheme that can be used in referring either to snow or to related notions such as ice or cold, as in Aztec (Nahuatl). This example implicitly appeals to the same causal logic introduced above. Here, the physical environment (a warm climate) shapes local cultural communicative needs (there is less need to communicate precisely and informatively about ice and snow than there would be in a colder climate), which in turn shape the category system of a language (allowing a broad and relatively uninformative semantic category encompassing both ice and snow). To our knowledge, this line of reasoning has not previously been tested broadly across languages.

## Analyses

To test this reasoning, we first asked whether the beginning and end points of the above causal chain—local environment and category system—are associated across languages in the manner predicted. We then asked whether the intervening variable, communicative need, also patterns as predicted by the causal chain.

We tested for an association between local environment and category system using two datasets. The first is based on a cross-language library survey of reference works covering a genetically diverse set of languages. For each language in our survey, we consulted a reference work for that language, and noted whether the same linguistic form appeared both as a translation of ice and as a translation of snow. For example, we found that in Tohono O’odham, a Uto-Aztecan language of southern Arizona, the same citation form *gew* is given as a translation of ice and a translation of snow [23]. The second dataset also provides translations in many languages but is based instead on two major online resources: the Intercontinental Dictionary Series (IDS) [24] and the World Loanword Database (WOLD) [25]. We refer to this as the IDS+ dataset after its primary source. The IDS+ dataset contains evidence confirming Whorf’s observation about Nahuatl: the same form in that language is provided as a translation of ice and a translation of snow, as with Tohono O’odham in the library dataset.

These two datasets have complementary strengths. The library dataset is smaller but seeks balanced coverage of the world’s language families. The IDS+ dataset is not balanced (for which we correct in our analyses) but is larger. For each language in each dataset, we determined the language’s canonical geographic location [26] and the mean temperature for that location. Full details of all analyses referenced here are provided below in the section on Materials and Methods.

If speakers of a language have never encountered snow, or mention of snow, that language is trivially unlikely to include a word for snow. Consistent with this prediction, initial analyses of both our datasets show that languages that lack a translation for ice or snow or both tend to be found in warmer regions. Although this result is consistent with the principle of efficient communication, it deserves little attention—it is no more interesting than the observation that Eskimo languages tend to lack words for kangaroos, or that Australian languages tend to lack words for polar bears.

**Fig 1:**
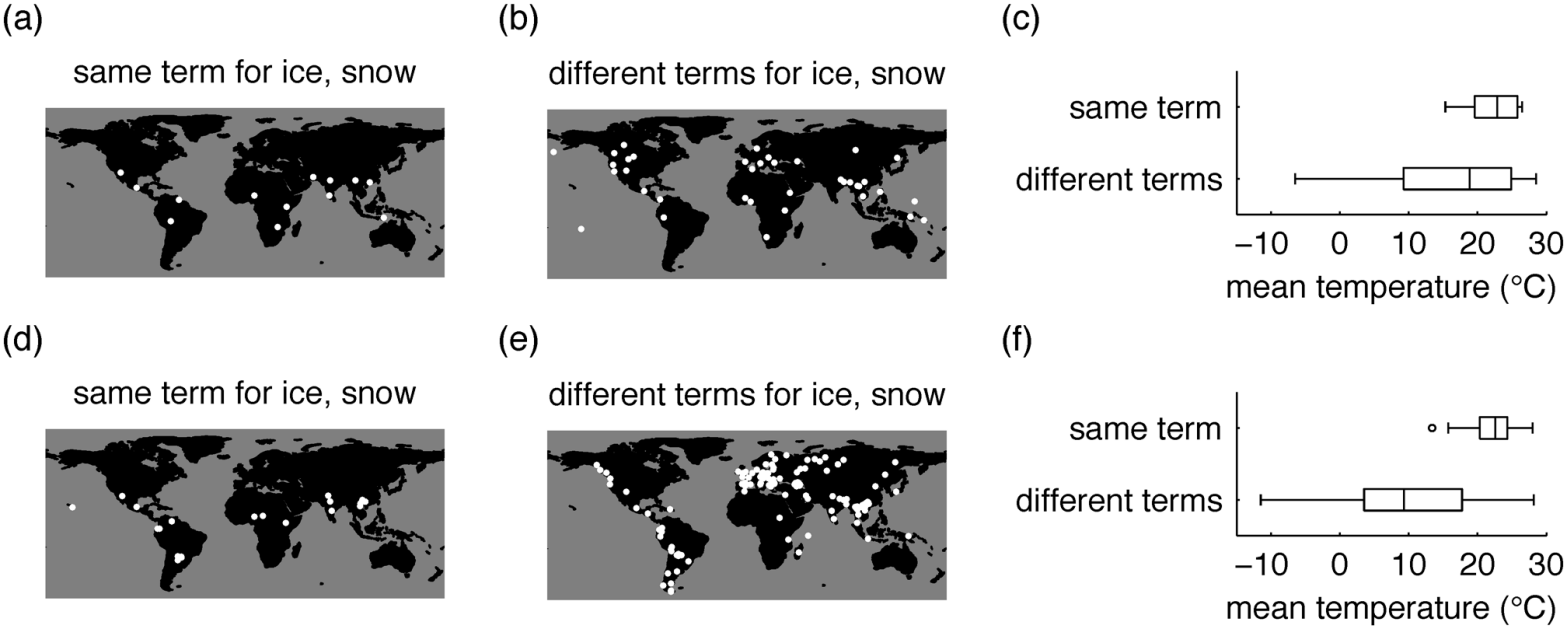
Results of cross-language surveys. Top panels: Library survey. (a) Locations associated with 13 languages that use the same term for ice and snow, and (b) 37 languages that use different terms. (c) Temperatures associated with the locations shown in (a) and (b). Bottom panels: IDS+ data. (d) Locations associated with 21 languages that use the same term for ice and snow, and (e) 145 languages that use different terms. (f) Temperatures associated with the locations shown in (d) and (e). Map data for this and all subsequent figures are from naturalearthdata.com.

For this reason, in our primary analyses, we considered only languages that include forms for both ice and snow, and asked for each language whether any form was used for both concepts. Working with these languages allowed us to focus on cultures that have some occasion to refer to both ice and snow, and allowed us to focus on the semantic breadth of forms for ice and snow, rather than on the presence or absence of such forms. The data in Fig 1 are drawn from these languages, and confirm the prediction that languages using the same term for ice and snow tend to be found in warmer locations. The more informative use of different terms for ice and snow is found in both warm and cool locations. This asymmetric pattern suggests a general preference for informative, precise communication, probabilistically modulated by local communicative need, as we discuss in greater detail below.

We analyzed the data in Fig 1 using two statistical approaches. We first ran a mixed effects logistic regression analysis of the relationship between temperature and use of the same term for ice and snow. To allow for genetic relatedness of languages, we included random intercepts for each language family. We found that temperature was positively associated with use of a single ice/snow term, as a fixed effect, in both the library dataset (*β* = .12, *χ*^2^(1) = 6.1, *p* = 0.013) and the IDS+ dataset (*β* = .18, *χ*^2^(1) = 18.2, *p* = 0.00002). The *β* values can be interpreted by computing the probability, according to these models, of a language having a single term for ice and snow at temperatures associated with English (8.7°C) and Nahuatl (21.8°C). These two probabilities are 0.09 and 0.31 in the library dataset, and 0.03 and 0.26 in the IDS+ dataset. We ran follow-up logistic regressions that controlled for areal relationships in addition to genetic relationships between languages, and found that the results for both datasets remained robust when language family and language area were both included as random effects. We then ran a second analysis to examine the asymmetric pattern evident in Fig 1—namely that merging ice and snow is associated with warm regions, but that distinguishing ice and snow is not especially associated with cold regions. We explored this idea using a Monte Carlo approach similar to one developed by Everett et al. [9], and the results provide statistical support for this asymmetry.

**Fig 2:**
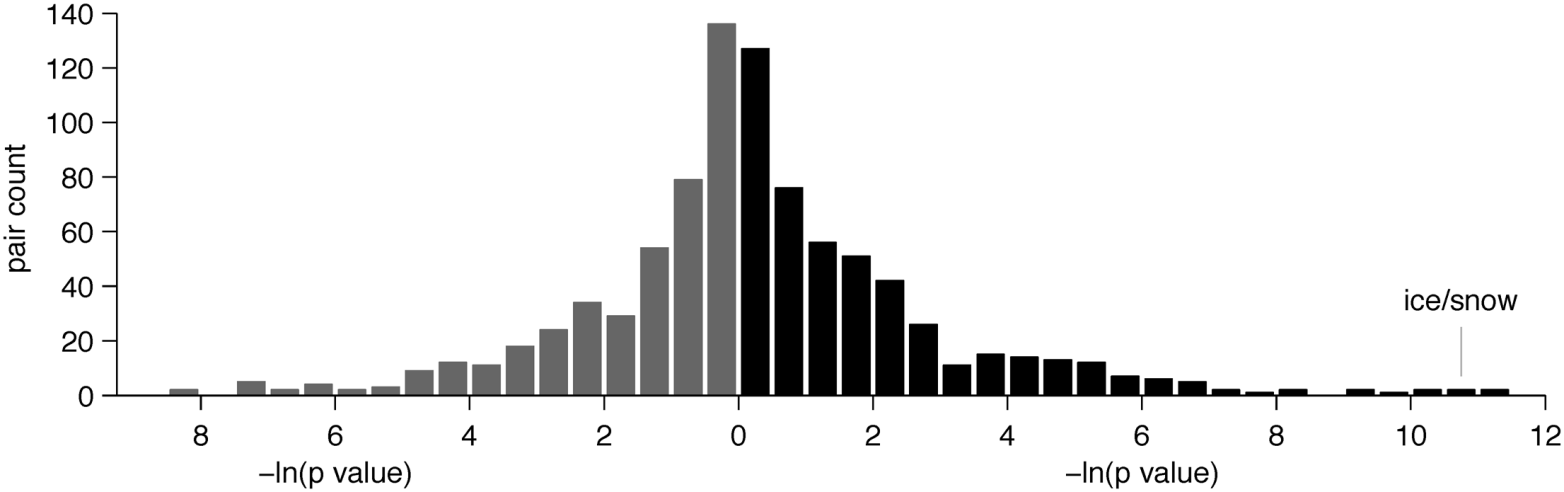
Results of an exhaustive analysis of meaning pairs in the IDS+ dataset. For each pair of meanings, we conducted a mixed effects logistic regression across languages, with mean temperature as the independent variable, and use of a single linguistic form for the two meanings as the dependent variable. For each pair, we compared models with and without temperature as a fixed effect, and recorded the *p* value associated with this comparison. Two histograms are displayed back-to-back: the gray histogram counts pairs for which the use of a single form is non-positively (zero or negative coefficient) associated with temperature, and the black histogram counts pairs for which the use of a single form is positively (positive coefficient) associated with temperature. The x-axis for each histogram shows minus natural log of the resulting *p* value, such that higher values correspond to greater significance; this quantity increases to the right for the positive histogram, and to the left for the non-positive histogram. The y-axis shows the number of IDS+ pairs for which that -ln(*p*) value was obtained.

The results of these analyses appear to support the causal chain presented above, but other explanations are possible. For example, it is known that complexity of the lexicon in several semantic domains correlates with societal complexity [27], and societal complexity tends to be lower in regions near the equator [28]. Thus, languages spoken in warm regions might tend to have fewer and broader semantic categories generally, not just for ice and snow. Moreover, the link between temperature and ice/snow might be only rather weakly significant relative to other comparable links in the same dataset. To address these concerns, we compared the IDS+ association between temperature and ice/snow with associations between temperature and other pairs of meanings that are named by the same linguistic form in some languages [29]. The IDS+ dataset is organized around a standard set of fine-grained meanings covering many aspects of human experience, and we considered all 935 pairs of meanings that are named by the same form in 10 or more of the languages of this dataset. For each such pair of meanings, we examined the association between local temperature and the use of a single form for that pair of meanings, across languages. Fig 2 shows that the statistical significance of the predicted positive relationship between temperature and ice/snow is extreme compared to results for other pairs of meanings [30]; the only two pairs that produce a more significant positive relationship are man/male (of an animal) and air/wind. Ice/snow is therefore one of the few instances of semantic breadth that emerge as especially significantly positively linked with temperature in an exhaustive search.

**Fig 3:**
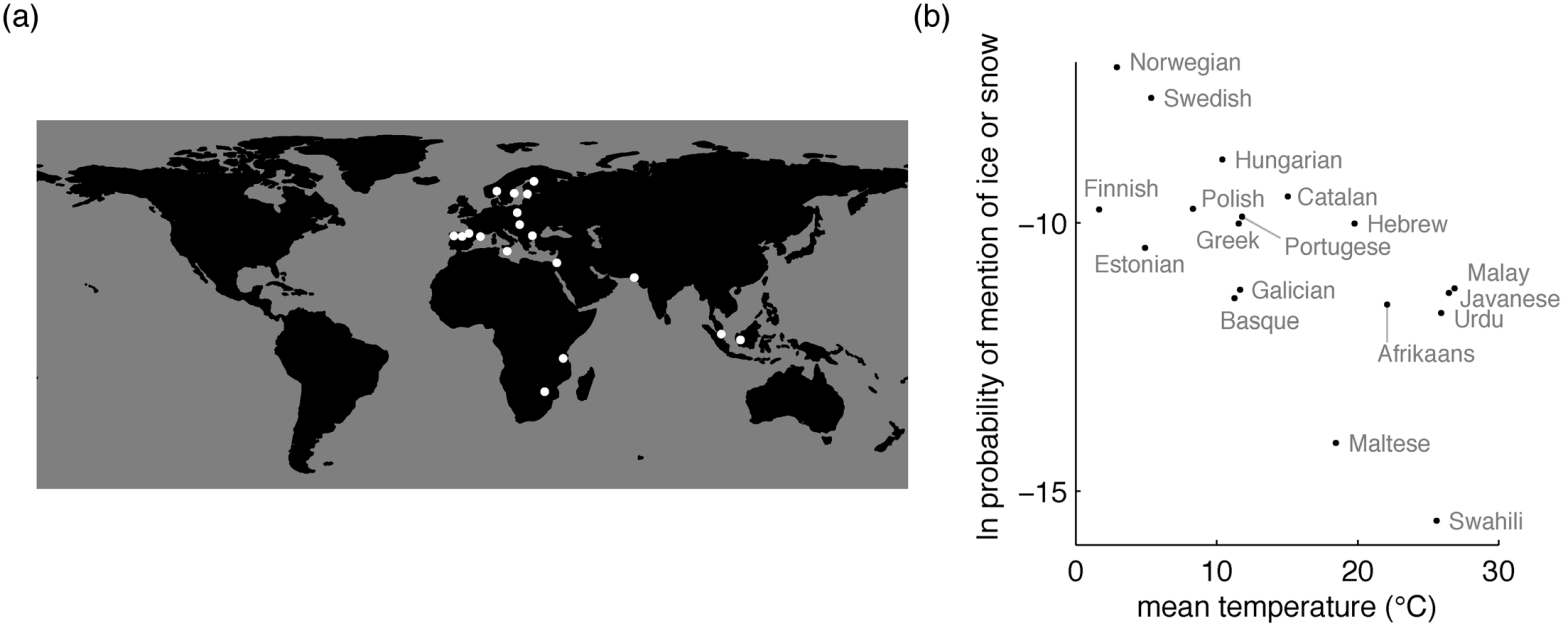
Results of cross-language Twitter analysis. (a) Locations associated with the 18 languages represented in the Twitter dataset we consider. (b) The natural log probability of mention of ice or snow in a given language as a function of the mean temperature where that language is spoken. Mixed effects logistic regression revealed that temperature is negatively associated with probability of mention of ice or snow, as a fixed effect (*β* = −0.29, *χ*^2^(1) = 21696, *p* < 10^−15^), when including random intercepts for each language family.

**Fig 4:**
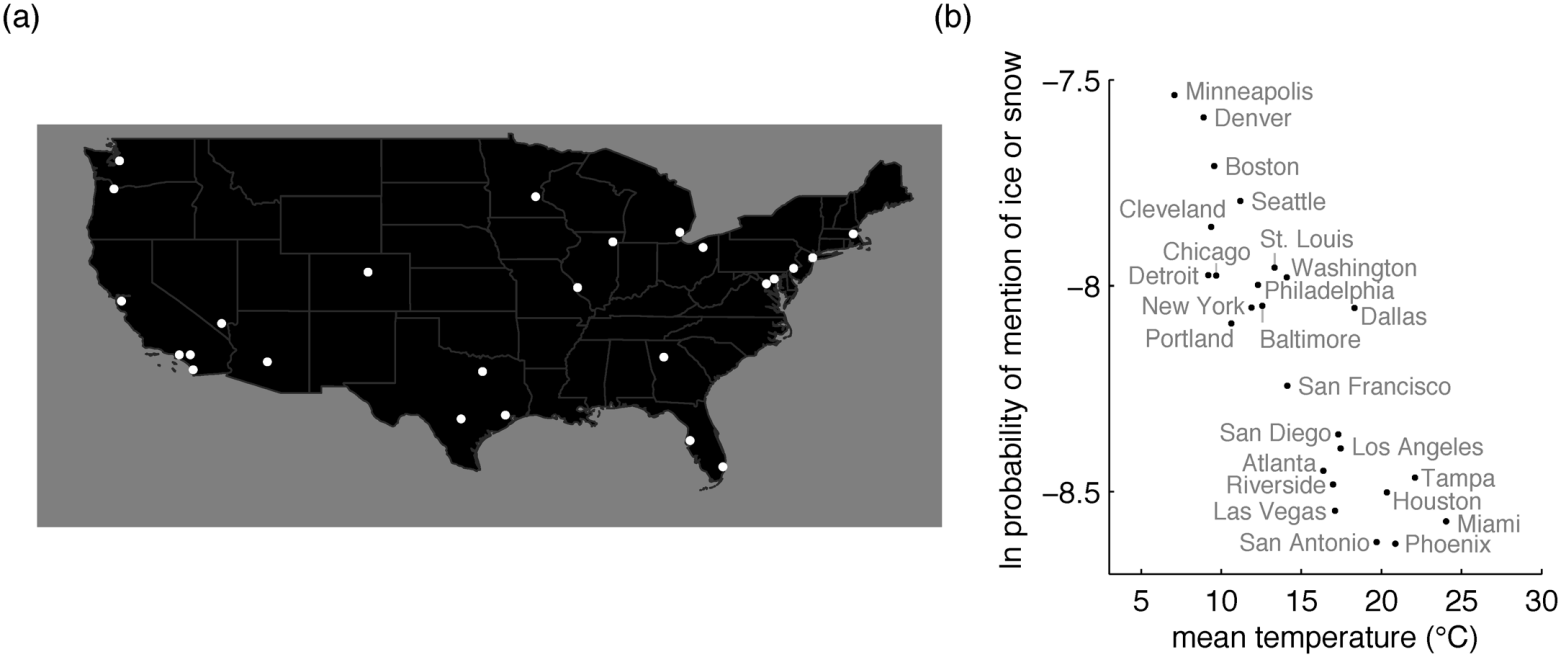
Results of US Twitter analysis. (a) Locations associated with the 25 urban areas represented in the Twitter analysis of American English. (b) Natural log probability of mention of ice or snow in a given urban area as a function of the mean temperature in that area. Logistic regression revealed that temperature is negatively associated with probability of mention of ice or snow (*β* = −0.060, *χ*^2^(1) = 322, *p* < 10^−15^).

The causal chain above holds that the link between temperature and existence of a single ice/snow term is mediated by the intervening variable of low communicative need to refer to ice or snow in warm climates. Intuition suggests that speech communities in cold climates should have greater need to refer to ice and snow than those in warm climates. However another possibility is that when snow is very common it may be taken as a “constantly assumed background” [4] to everyday life and therefore explicitly mentioned only rarely. We directly probed communicative need to refer to ice and snow as a function of local temperature, by examining a large multilingual dataset of messages posted to Twitter between 2009 and 2013 [31]. We chose the Twitter dataset for this purpose because Twitter messages include broad-ranging comments about the state of the world, and word frequencies in these messages can therefore be taken as a rough proxy for local communicative need. For each language represented in the Twitter dataset, we noted: (1) the number of uses in that language of the form or forms that Google Translate provides as that language’s translations for English *ice* and *snow*, (2) the number of uses in that language of all other forms, so that usage of forms for ice or snow can be considered as a proportion of all usage, and (3) the mean temperature for the geographic location associated with that language. Fig 3 shows that the log of the probability of mention of ice or snow is negatively associated with temperature. This finding suggests that speakers of languages associated with warm climates do tend to mention ice and/or snow proportionally less than speakers of languages associated with cold climates. This analysis is based on citation forms (e.g. Swedish *snö*, “snow”), and does not incorporate counts for inflected forms (e.g. Swedish *snön*, “the snow”). Languages vary in their inflectional morphology, so we supplemented this analysis with another that analyzes usage frequency among speakers of a single language. Fig 4 shows that the same negative relationship between temperature and usage also holds among speakers of English across the continental United States. The results of both Twitter analyses are consistent with the logic of the causal chain above, as they suggest reduced pressure for precise communication about ice and snow in warm climates, and greater pressure for such communication in cold climates.

## Discussion

The notion of “words for snow” has occupied an uncomfortable position in the debate over language and cognition. Apparently initially intended as a simple illustration of the fit of semantic categories to local needs, it has been much exaggerated, and in that distorted form has become a prominent cultural reference point. In turn, that popular distortion has itself become the target of critique [3] and parody [4]. In an influential if tongue-in-cheek essay, Pullum [4] dismissed the entire topic as an urban legend: “the great Eskimo vocabulary hoax”. Pullum highlighted in particular the extravagant numbers of words for different types of snow that some have attributed, with little or no evidence, to Eskimo languages. We agree that this numerical inflation deserves to be lampooned. However, we disagree with a separate point that Pullum advances in the same essay:

[E]ven if there were a large number of roots for different snow types in some Arctic language, this would not, objectively, be intellectually interesting; it would be a most mundane and unremarkable fact. … Horsebreeders have various names for breeds, sizes, and ages of horses; botanists have names for leaf shapes; interior decorators have names for shades of mauve; printers have many different names for different fonts … If these obvious truths of specialization are supposed to be interesting facts about language, thought, and culture, then I’m sorry, but include me out.

(p. 165)

The idea that languages are adapted to the needs of those who speak them is indeed intuitively natural, and this naturalness may account in part for the popularity of the snow example. But the fact that an idea is intuitively natural does not make it trivial. Intuitions can be misleading, and it is important to test them. Moreover, it is clear that for professional reasons horsebreeders, botanists, interior decorators, and printers need to attend to fine-grained distinctions among horses, leaves, colors, and fonts respectively—so their fine-grained semantic divisions of those domains are indeed unsurprising. But as Pullum himself suggests elsewhere in the essay, the Eskimo case need not be parallel:

Eskimos aren’t really that likely to be interested in snow. Snow in the traditional Eskimo hunter’s life must be a kind of constantly assumed background, like sand on the beach. And even beach bums have only one word for sand.

(p. 166)

Do those who live surrounded by snow attend closely to it—as a horsebreeder does to horses—or do they mentally background it so as to attend closely to other things—as a beach bum presumably backgrounds sand? Given these conflicting intuitions, the local communicative importance of this notion would need to be empirically assessed, as we have attempted to do in our Twitter analyses.

There are at least two additional reasons why the topic of words for snow deserves detailed empirical investigation. First, it provides a counterpoint to the recent claim of cross-language universals in lexical semantics that are largely independent of geography and environment [32]. Second, and perhaps most importantly, something larger is implicated here: the general theoretical proposal that language is shaped by the need for efficient communication. Several aspects of language and language use have been explained in terms of this proposed need [11–21], and we have argued that Boas’ original snow claim is an instance of this same principle. It is in fact an especially high-profile instance—so if the line of reasoning behind it had turned out to be empirically unsupported, that would have been damaging to an influential and broadly referenced theoretical proposal. For these reasons we consider the question to be a significant one.

As it happens, a recent study has argued that Boas’ original claim was in fact correct. Krupnik and Müller-Wille [5] have argued, contra Pullum, and on the basis of several empirical datasets, that “the English vocabulary for snow and related phenomena is clearly inferior to those recorded in several Eskimo/Inuit languages and dialects” (p. 391). They argue further that this phenomenon is not limited to Eskimo/Inuit languages, but also extends to other languages spoken in cold climates where snow is common, such as Russian. They illustrate this point with several Russian lexemes, including one that interestingly captures the absence of snow where it might be expected: “*protalina* (open ground where the snow has melted)” (p. 394). Finally, they suggest that the entire debate has been somewhat empirically misdirected, in that Eskimo languages tend to exhibit a richer vocabulary for types of sea ice than for types of snow—and that a truly rich snow vocabulary may be found elsewhere, among the Norwegian Sámi.

Our goal here has been to broaden this debate beyond Eskimo languages, and thereby to re-focus attention on the general principle Boas appears to have originally had in mind, rather than the specific example he used to illustrate it. That principle is that the lexicon of a language reflects local communicative needs, and may therefore be shaped by the local environment. Importantly, whether or not that principle is clearly supported in the specific case of Eskimo/Inuit languages, the more general question would remain unsettled: one could always reasonably argue that the Eskimo case might be an unrepresentative statistical outlier. The general question concerns the relation of language and the environment, and the universal scope of that question requires that it be tested using as broad a range of languages as possible. For that reason, we have focused not on Boas’ original proposal of a link between cold temperature and fine-grained semantic subdivision of the notion of snow, but instead on Whorf’s inverse application of the same principle, predicting a link between warm temperature and a single semantic category encompassing both ice and snow. That instantiation of the principle can be tested using a wide range of languages across the earth’s surface, unlike Boas’ original formulation which requires an empirical focus near the polar regions.

When considering the distribution of terms for ice and snow across the world’s languages, two naturally opposed if somewhat simplified stances suggest themselves, as a means of framing the question. A strict universalist stance would hold that ice and snow are fundamental concepts that should be lexically distinguished in all languages. In contrast, a strict environmental determinist stance would hold that these two notions should always be treated differently in cold vs. warm regions; for example, that these two notions should always be lexically distinguished in cold regions, and not distinguished in warm regions. Our results partially match the predictions of each of these idealized stances, yet perfectly match the predictions of neither. We find that languages with separate terms for ice and snow are spoken in both cold and warm regions—whereas languages that collapse this distinction in their lexicons are spoken exclusively in warm regions. This asymmetrical pattern in the data may be attributable in part to languages in warm regions sometimes being culturally influenced by those from colder regions, for instance through colonialism or the spread of industrialization. In fact, several of the languages in our data that are spoken in warm regions (e.g. Kâte) preserve the ice/snow distinction through what appear to be borrowings from English or another colonial language—although other “warm” languages associated with a history of colonial rule (e.g. Hindi) collapse the distinction. But this asymmetry is also consistent with a view in which language is probabilistically shaped by the need for efficient communication. If warm surroundings decrease the communicative need to refer to ice and snow, that decreased need would reduce communicative pressure to preserve the ice/snow distinction, and we would expect to see the ice/snow distinction collapsed more often in warm regions than in cold ones. Importantly, the causal links in this account are probabilistic, not deterministic. Thus, there is no prediction that *all* languages in warm regions will necessarily collapse the ice/snow distinction—just that there should be less pressure to preserve the distinction, and thus a stronger tendency to collapse it, in warm than in cold regions. Our data are consistent with that prediction.

To our knowledge, this connection between temperature and ice/snow terminology has not previously been reported. Some of our analyses relied on large online datasets that have only recently become available, and thus these analyses could not have been conducted much earlier. However, a library survey like ours could have been conducted some time ago, and if it had, presumably the same pattern would have been found. If we are right that this has not previously been done, why not? We cannot be certain, but one possible reason is connected with the asymmetrical pattern in our data that we have just discussed. It is very easy to find languages in warm regions that preserve the ice/snow distinction, and if one is thinking in deterministic rather than probabilistic terms, such examples could be taken as simple falsification of the claim. It is only when allowing a probabilistic view of the connection between language and the environment that the general picture we have reported here emerges.

## Conclusions

Our results support the claim that local communicative needs can leave their imprint on category systems across languages, a point that may generalize across semantic domains. They also suggest that this claim can be viewed as an instance of the more general theoretical stance that language reflects the need for efficient communication. We hope that our results will help reclaim for analytical study an important topic that has been to some extent lost to popularization. We also hope our results will help to rehabilitate a widely criticized yet sensible way of thinking about variation in semantic systems across languages.

## Materials and Methods

Code and data supporting the analyses reported here are available at https://github.com/cskemp/icesnow.

### Nomenclature

We understand that the term “Eskimo” is considered derogatory in some locales, and not in others (https://www.uaf.edu/anlc/resources/inuit-eskimo/). In using the term here, we certainly do not intend any derogatory meaning. We also feel it would be confusing to avoid the term when the debate to which we respond has used it so extensively.

### Library survey

#### Reference works considered, and data extraction

We considered language reference works (dictionaries, word lists, or grammars that supported a lookup of lexical items) that were available in the UC Berkeley library, and that were written either in English or in another language that is accessible to at least one member of our research team, e.g. Spanish or French. We sought translations for ice and snow in these reference works. Some references contained blank entries as translations for either ice or snow, suggesting that that language had been found to lack a form expressing that idea. We considered a reference work to contain *appropriate documentation* if it included a non-blank entry as a translation for ice and a non-blank entry as a translation for snow. For each reference work that contained appropriate documentation, we noted the full list of translations given for ice, and the corresponding full list given for snow. If there was a single linguistic form that appeared as a translation in both lists, we considered that language to use the same form for ice and snow; otherwise we considered that language to use different forms for ice and snow. Many reference works included disambiguating information (e.g. snow as noun rather than verb, or as a form of frozen water rather than a colloquial term for cocaine); however some did not. We assumed that ambiguous entries corresponded to nouns with our target meaning.

#### Sampling procedure

We sought to obtain a genetically diverse language sample, with data for each language in the sample. To that end, we followed as closely as possible an existing cross-language study by Bybee et al. [33]. They constructed a language survey with entries for 94 languages, distributed across language families, following the language classification scheme of Voegelin and Voegelin [34]. Each entry was intended to represent a particular Voegelin and Voegelin language group, so they sought to populate each entry with a single language from the corresponding group. Their resulting sample included languages for 76 of these entries, because they were unable to find data from languages in the groups corresponding to the other 18 entries. We sought to include in our sample the 76 languages that Bybee et al. listed in theirs, with the addition of one language for each entry that remained unpopulated in their sample.

We began by searching for reference works for each of the languages in the Bybee et al. sample. If we were unable to find appropriate documentation (as defined above) for one of these languages, we searched in random order through the other languages in the corresponding Voegelin and Voegelin group until we either found a reference work that contained appropriate documentation, or exhausted the list of languages in the group. For each of the 18 entries for which Bybee et al. did not find data, we searched in random order through all languages in the corresponding Voegelin and Voegelin group until we either found a reference work that contained appropriate documentation, or exhausted the list of languages in the group.

For each target language, we searched the UC Berkeley library catalog for that language name, and all alternate language names supplied for that language by Voegelin and Voegelin (1977) [34]. We retrieved each reference work found in that search that met our criteria (written in a language accessible to us, etc.), and determined whether it contained appropriate documentation; if so, we terminated the procedure for that language at that point. Thus, we included in our dataset data from the first reference work on the first language in its group that contained appropriate documentation. If we exhausted all reference works for a given target language without finding one that contained appropriate documentation, we determined that there was not appropriate documentation available for that language. If all languages in a group lacked appropriate documentation, we did not include a language from this group in our sample.

#### Data

The results of this survey are listed in S1 Table of the Supplementary Information. We were able to find appropriate documentation for 50 of the 94 language entries.

#### Glottocode lookup

For each language in our sample, we sought to determine its Glottocode (for use in Glottolog) by looking up that language’s reference work in Glottolog and using the Glottocode associated with that reference. This yielded unambiguous results for 37 of the 50 library survey languages. For the remainder, the Glottolog database either did not include the target reference work, or associated it with the Glottocode for some other language; in these cases we searched for Glottolog references associated with the language name, and used the Glottocode associated with the plurality of these references. We verified that all references being considered were for languages located relatively near the target language.

### IDS+ data

The IDS+ dataset is based on wordlists from the Intercontinental Dictionary Series (IDS) [24] and the World Loanword Database (WOLD). The IDS includes 288 wordlists in total, and we dropped 4 lists that were not associated with ISO language codes. In addition we dropped the list for Elamite because it seemed to include forms for multiple variants of Elamite, and the list for Khasi, which was empty, leaving 282 wordlists. WOLD includes 41 wordlists and we used all of them.

Some of the IDS+ wordlists are associated with the same ISO language code—for example, both WOLD and the IDS include wordlists for English. 246 distinct languages (i.e. 246 distinct ISO codes) are represented among the full set of 323 wordlists. Our analyses were therefore conducted on a per-language, rather than per-wordlist, basis.

Each wordlist includes linguistic forms for a subset of 1310 standard meanings. In some cases there are multiple forms for a given meaning, and in other cases no form is provided. We handled both situations using the same approach used for the library survey. A language represented by multiple lists was deemed to use the same form for ice and snow if any of these lists included identical forms for ice and snow.

As part of a previous study of semantic categories across languages, List et al. [35] developed a database of Cross-Linguistic Colexifications (CLICS) using wordlists from online resources including IDS and WOLD. Our code for processing wordlists is based in part on the CLICS codebase.

#### Data

Among the 246 IDS+ languages, we found that 21 had the same form for ice and snow. These languages are: Cofan, Ecun Buyang, Gawwada, Guarani, Hausa, Hawaiian, Hindi, Imbabura Quechua, Kanuri, Maca, Mocovi, Mulam, Nahuatl, Nung-Ninbei, Panare, Pilaga, Punjabi, Qau Kelao, Telugu, Toba, and Yaqui.

### Temperature data

Our temperature variables are drawn from the CRU Global Climate Dataset. We used a version of the data [36] that includes average monthly values for the period 1961–1990. The data set includes environmental variables that are specified over a grid that covers global land areas (excluding Antarctica) at a resolution of 0.5° latitude by 0.5° longitude. One such environmental variable is mean monthly temperature. For each geographic location in the data set, we averaged this quantity over the 12 months of the year to obtain an estimate of the mean temperature for that location.

Each language in our data sets was linked with a geographic location using latitude and longitude information from version 2.4 of Glottolog [37]. Latitudes and longitudes were not available for 10 of the 246 IDS+ languages, leaving 236 that were used for subsequent analyses.

### Languages missing forms for ice, snow or both

Although our primary analyses focus on languages that include forms for both ice and snow, we also tested the prediction that languages missing one or both of these forms should tend to be found in warmer regions. In the library dataset, as noted above, there were several language groups for which we failed to find any reference work for any language in that group that contained translations for both ice and snow. These groups fell predominantly in the Andean-Equatorial, Australian, Austronesian, Indo-Pacific, and Niger-Kordofanian maximal language groupings of Voegelin and Voegelin—and these maximal language groupings are largely located in warm regions.

**Fig 5:**
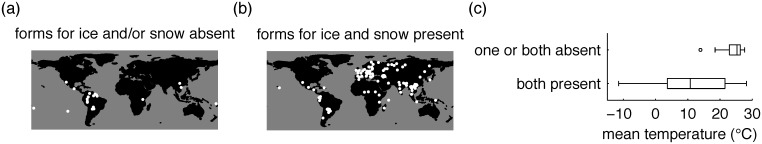
Analysis of missing forms in the IDS+ data. Locations associated with (a) 20 languages that are missing forms for ice or snow or both and (b) 140 languages that include forms for both meanings. (c) Temperatures associated with the locations shown in (a) and (b).

The IDS+ dataset is larger than the library dataset, and contains more information for each language; thus we conducted a more formal analysis on it. Some languages may be missing terms for ice and or snow because they are sparsely documented in general. When analyzing missing terms we therefore considered only IDS+ languages that include forms for 1000 or more of the 1310 meanings in our standard list. Among these languages, 20 lacked forms for either ice, snow or both (Fig 5a) and 140 included forms for both ice and snow. Fig 5c confirms that languages missing one or both forms tended to be found in warmer regions. A mixed-effects logistic regression revealed that temperature is positively associated with missing forms for ice and/or snow, as a fixed effect (*β* = 0.21, *χ*^2^(1) = 20.1, *p* < 10^−5^), when including random intercepts for each language family.

### Mixed-effects logistic regression analyses

All mixed-effects logistic regressions were carried out in R using the glmer() function. When analyzing the data in Fig 1, temperature was the independent variable and the dependent variable indicated whether or not a language uses the same term for two meanings (ice and snow). To allow for relationships between languages we included a random intercept for each Glottolog language family. We did not include random slopes because in the library dataset the number of language families is relatively large with respect to the number of languages. We tested the relationship between the independent and dependent variables for significance using a likelihood ratio test to evaluate the improvement achieved when temperature is included as a fixed effect relative to the reduced model that does not include temperature.

The analyses that generated Fig 2 repeated the procedure just described for 935 dependent variables that indicated whether or not languages use the same term for 935 different pairs of meanings. These 935 pairs included all pairs of meanings that are associated with the same form in at least 10 of the IDS+ languages. Logistic regressions for 36 of the pairs did not converge, and Fig 2 is based on the remaining 899 pairs. When analyzing each pair of meanings, we dropped languages that did not include forms for both meanings. As a result, analyses of different pairs are typically based on different subsets of the IDS+ languages.

**Fig 6:**
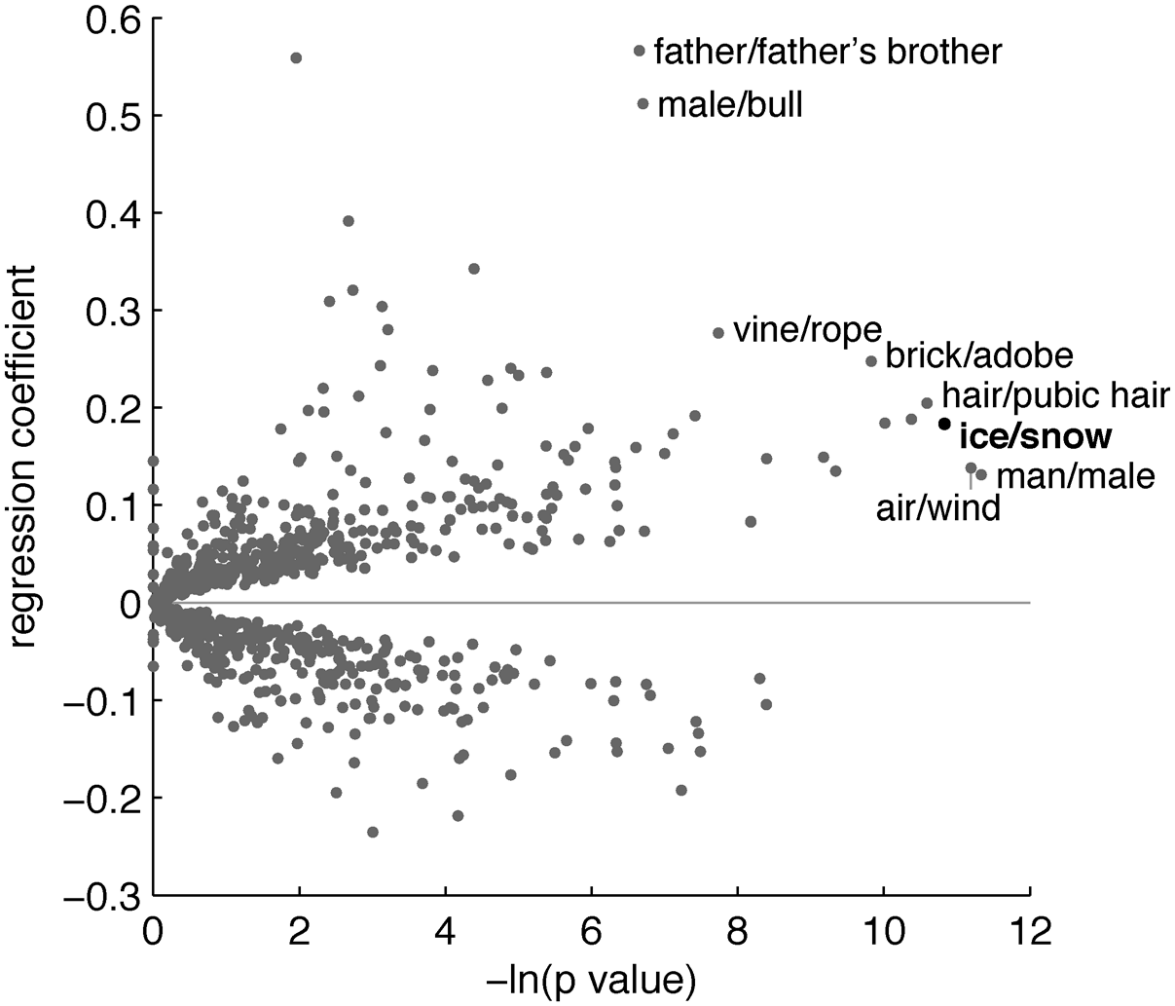
Regression coefficients and -ln(*p*) values for the exhaustive analysis of meaning pairs in the IDS+ dataset. Labels are included for all non-dominated pairs that are associated with a positive regression coefficient. Two of these pairs include “male,” which is a meaning associated with animals rather than people.

Fig 2 focuses on *p* values that characterize the statistical significance of relationships between temperature and use of the same form for meaning pairs, but the strength of these relationships is also important. Fig 6 provides a more complete picture by including the regression coefficient and *p* value for each meaning pair. The coefficients for some pairs (e.g. vine/rope) exceed that of ice/snow, but in all such cases ice/snow has the smaller *p* value. Ice/snow is therefore one of 8 pairs that are non-dominated in this sense.

### Areal relationships between languages

To control for areal as well as genetic effects we used mixed-effects logistic regressions that included both language area and language family as random effects. Language areas were drawn from the AUTOTYP database [38], and we mapped Glottocodes on to AUTOTYP LIDs using a table included in the lgfam-newick repository [39]. If a Glottocode was associated with multiple LIDs, we used the LID most frequently associated with the Glottocode. In a handful of cases two LIDs were associated with a Glottocode equally often, and we resolved all ties using our judgment about the languages in question.

Starting with the model that includes a random intercept for language family, likelihood ratio tests suggest that adding a random intercept for language area does not significantly improve model fit for either the library (*χ*^2^(1) = 0, *p* = 1) or IDS+ datasets (*χ*^2^(1) = 1.38, *p* = 0.24). If we include an intercept for area anyway, temperature remains significantly associated with use of a single term for ice and snow, in both library (*β* = 0.13, *χ*^2^(1) = 6.41, *p* = 0.01) and IDS+ datasets (*β* = 0.19, *χ*^2^(1) = 13.4, *p* = 0.0002). Similar results emerge if we include both an intercept and a slope for language area (library: *β* = 0.13, *χ*^2^(1) = 2.10, *p* = 0.036; IDS: *β* = 0.19, *χ*^2^(1) = 12.3, *p* = 0.0005).

### Monte Carlo analysis of asymmetry

The box-and-whisker plots in Fig 1c and 1f represent one of three ways in which the hypothesis of efficient communication could be supported. We will refer to these three possibilities as *no overlap*, *warm lumpers*, and *cool splitters.* *No overlap* is the case in which splitting languages (those with different terms for ice and snow) tend to be found in cool regions, and lumping languages (those with the same term for ice and snow) tend to be found in warm regions. In this case the boxes for splitters and lumpers would not overlap, or would overlap only slightly. *Warm lumpers* is the case in which splitting languages are found in both cool and warm regions, but lumping languages are found in warm regions only. *Cool splitters* is the parallel case in which splitting languages are found only in cool regions, but lumping languages are found in both cool and warm regions.

**Fig 7:**
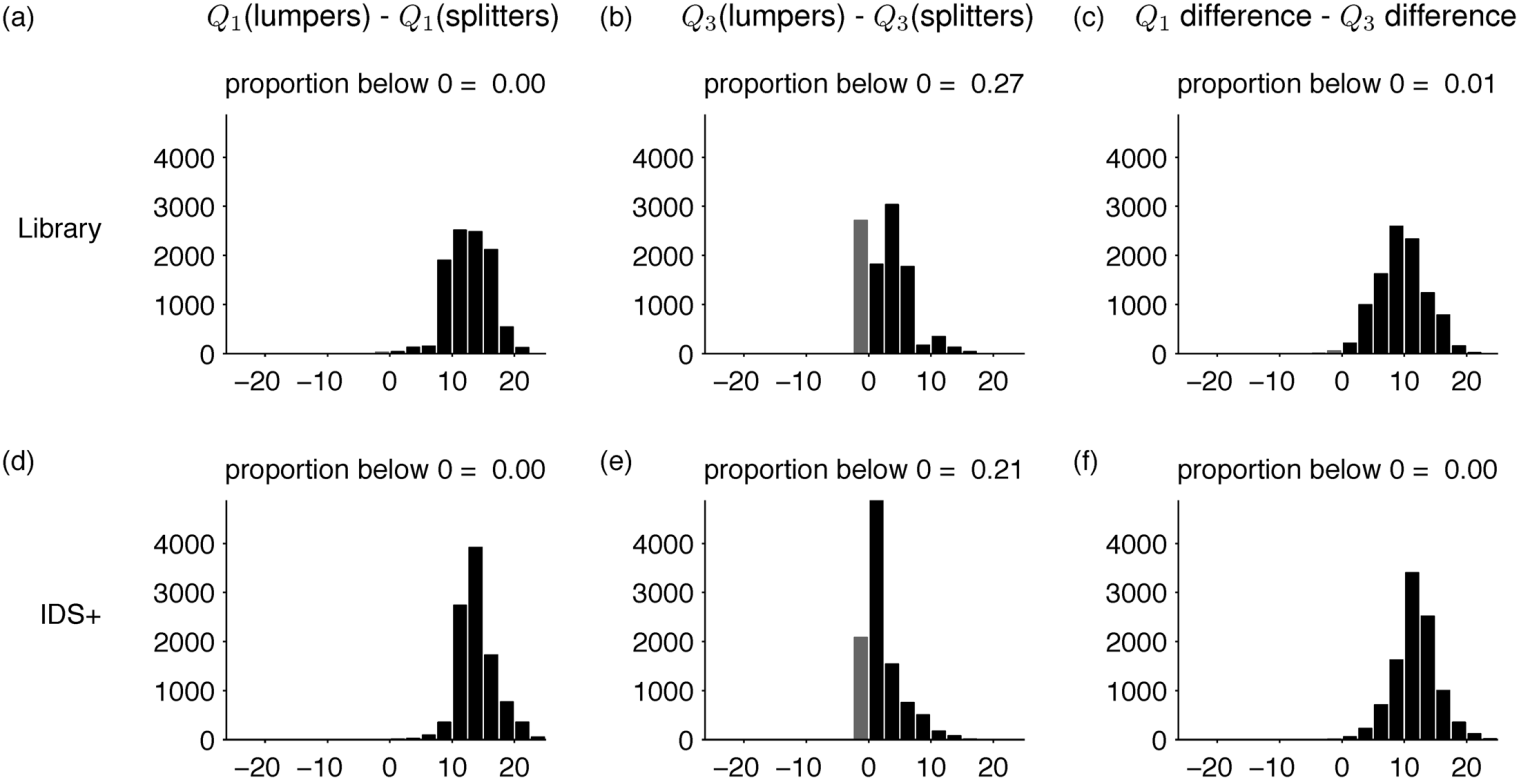
Monte Carlo results. The first column shows that the first (lowest) temperature quartile of a lumper sample is almost always higher than the first quartile of a splitter sample. The second column shows that the differences between third quartiles of the samples are less skewed. The third column confirms that differences between the first quartiles are greater than differences between the third quartiles. Results for the library and IDS+ datasets are shown in the first and second rows respectively. The x axis in each plot shows differences in degrees Celsius, and the y axis shows number of samples.

To confirm that our data represent an instance of *warm lumpers*, it is necessary to control for language relatedness. We did so using a Monte Carlo analysis in which we repeatedly sampled subsets of the lumpers and splitters (cf. [9]). Each sample of lumpers included one language from each language family represented among the lumpers. Each sample of splitters matched the lumper samples in size, and included at most one language from each family represented among the splitters. Comparing the quartiles of the temperatures associated with lumper and splitter samples provides a way to distinguish among the three possibilities mentioned above. Fig 7a and 7d show that the first (lowest) quartile of the lumper temperatures is almost always higher than the first quartile of the splitter temperatures. Fig 7b and 7e show that the corresponding result based on third quartiles is weaker. The “difference of differences” plots in Fig 7c and 7f confirm that the difference in temperatures is more extreme for first quartiles than for third quartiles. These results are more compatible with “warm lumpers” than with the other two possibilities.

### Cross-linguistic Twitter analysis

Our frequency analyses used the Gardenhose/Decahose stream from Twitter, which includes around 10% of all public tweets. Our cross-linguistic analysis used all Gardenhose/Decahose tweets posted between January 1, 2009 and December 31, 2013. We used langid.py [40] to identify the language in which each tweet was written. This package provides a confidence score for each classification, and we dropped all tweets that were classified with confidence less than 90%. Using the remaining tweets we compiled frequency statistics for the 42 languages with the most word tokens over our 5 year period. Two of these languages are Latin and Esperanto, and we dropped these languages from our analysis.

For each remaining language we used Google Translate to identify linguistic forms for ice and snow in that language. Breton and Aragonese are not currently supported by Google Translate so we dropped these languages. We also dropped Japanese, Thai, and Chinese because texts in these languages typically do not include spaces between words, making it difficult for us to compile token frequencies for these languages. 35 languages remained after this initial filtering process. Token frequencies for ice and snow forms were compiled for all 35 languages that remained.

Frequencies for some of these 35 languages are noisy because forms for ice and snow are often polysemous. For example, the Vietnamese form given for ice is *băng*, but the same form is also used for other meanings. To reduce the impact of polysemy, we removed all languages for which the forms for ice and snow generated English words other than *ice* and *snow* when submitted to Google Translate. For example, Vietnamese *băng* fails this back-translation test because English *band* is also generated as a possible translation. Fig 3 in the main text includes all 18 languages that passed the back-translation test.

To determine how heavily the result in Fig 3 is influenced by the back-translation test, we re-ran the analysis without including this test. Using all 35 languages described earlier, a mixed-effects logistic regression again reveals that temperature is negatively associated with probability of mention of ice or snow (*β* = −0.21, *χ*^2^(1) = 255374, *p* < 10^−15^).

### American English Twitter analysis

Our second Twitter analysis focused on tweets associated with the 25 largest US urban areas, as determined from the 2010 US census. Latitudes and longitudes for these areas were taken from the 2010 Urban Areas Gazetteer File [41].

A subset of the tweets in the Gardenhose/Decahose stream include geocodes. Twitter added official support for Geocodes in 2009, and we therefore worked with tweets posted between January 1, 2010 and December 31, 2013. Fig 4 of the main text is based on all tweets that had a geocode that fell within 50 miles of one of the 25 largest urban areas, and that were written in English according to langid.py.

Because the analysis focused exclusively on English, including language family as a random effect was not necessary. The results reported in the caption of Fig 4 are therefore based on a standard logistic regression conducted using R’s glm() function.

## Supporting Information

S1 Table References and data from the library survey. (PDF)
