# Supplementary material for: Languages Support Efficient Communication about the Environment: Words for Snow Revisited
Source: PLoS One. 2016 Apr 13;11(4):e0151138. doi: 10.1371/journal.pone.0151138 (PMC4830456; doi:10.1371/journal.pone.0151138)
Supplement: S1 Table — (PDF) [file pone.0151138.s001.pdf]

Supplementary Information

Table S1: **Library survey.** IDs and groupings are from Bybee et al., 1994. Form: S=same, D=different.

ID	Grouping	Language	Reference	Form
1	Unaffiliated	Inuit	Fortescue, M., Jacobson, S. A., & Kaplan, L. (1994). *Comparative Eskimo dictionary: with Aleut cognates.* Fairbanks: Alaska Native Language Center.	D
2	Unaffiliated	Basque	Aulestia, G. & White, L. (1990). *English-Basque dictionary.* Reno: University of Nevada Press.	D
4	Afroasiatic: Chadic: West Chadic	Hausa	Newman, R. M. (1990). *An English-Hausa dictionary.* New Haven: Yale University Press.	S
5	Afroasiatic: Other Afroasiatic	Oromo	Muudee, M. H. (1995). *Hamid Muudee's Oromo dictionary: English-Oromo.* Atlanta, GA: Sagalee Oromoo Publishing Company.	S
6	Afroasiatic: Other Afroasiatic	Kabyle	Dallet, J. (1982). *Dictionnaire kabyle-français.* Paris: SELAF.	D
8	Afroasiatic: Other Afroasiatic	Tigrinya	Aressi, T. (1987). *Concise English-Tigrinya dictionary.* Asmara: Ethiopian Studies Centre.	D
9	Macro-Algonquian	Cheyenne	Petter, R. (1915). *English-Cheyenne dictionary.* Kettle Falls, WA: Mennonite Mission.	D
12	Andean-Equatorial: Equatorial: Other Equatorial	Chacobo	Zingg, P. (1998). *Diccionario chácobo-castellano, castellano-chácobo.* La Paz: Confederación de Pueblos Indígenas de Bolivia.	S
22	Austroasiatic: Mon-Khmer: Bahnaric	Lue	Gedney, W. J. (1996). *William J. Gedney's The Lue language.* T. J. Hudak (Ed.). Ann Arbor: Center for South and Southeast Asian Studies, University of Michigan.	D
23	Austroasiatic: Mon-Khmer: Other Mon-Khmer	Khmer	Keesee, A. P. K. (1996). *An English-spoken Khmer dictionary.* London: Routledge.	D
24	Austroasiatic: Other Austroasiatic	Santali	Campbell, A. (1899). *A Santali-English dictionary.* Pokhuria, India: Santal Mission Press.	D
25	Austronesian: Oceanic: Eastern Oceanic	Tahitian	Wahlroos, S. (2002). *English-Tahitian, Tahitian-English dictionary.* Honolulu: Mā'ohi Heritage Press.	D

Table S1: (continued)

ID	Grouping	Language	Reference	Form
33	Austronesian: Oceanic: Other Oceanic	Trukese	Goodenough, W. H. & Sugita, H. (1990). *Trukese-English dictionary.* Philadelphia: American Philosophical Society.	D
34	Austronesian: Malayo-Polynesian	Pangasinan	Benton, R. A. (1971). *Pangasinan dictionary.* Honolulu: University of Hawaii Press.	D
36	Austronesian: Malayo-Polynesian	Tetun Dili	Williams-van Klinken, C. (2003). *Tetun language course.* Dili, East Timor: Peace Corps East Timor.	S
38	Aztec-Tanoan	Tohono O'odham	Saxton, D. & Saxton, L. (1969). *Dictionary: Papago & Pima to English, English to Papago & Pima.* Tucson, AZ: The University of Arizona Press.	S
39	Caucasian	Chechen	Awde, N. & Galaev, M. (1997). *Chechen-English English-Chechen dictionary and phrasebook.* New York: Hippocrene Books.	D
40	Macro-Chibchan: Chibchan	Kuna	Erice, J. (1985). *Diccionario de la lengua kuna.* Panama: INAC.	D
41	Macro-Chibchan: Other Macro-Chibchan	Warao	Barral, B. M. (1979). *Diccionario warao-castellano, castellano-warao.* Caracas: Universidad Católica Andrés Bello.	S
42	Dravidian	Badaga	Hockings, P. & Pilot-Raichoor, C. (1992). *A Badaga-English dictionary.* Berlin: Mouton de Gruyter.	S
45	Ge-Pano-Carib	Shipibo-Conibo	Loriot, J., Day, D., & Lauriault, E. (1993). *Diccionario shipibo-castellano.* Yarinacocha, Perú: Ministerio de Educación.	D
46	Hokan	Chumashan	Henshaw, H. W. (1884). *Chumashan and Costanoan vocabularies.* Washington, DC: Smithsonian Institution, Bureau of American Ethnology.	D
47	Indo-European: Italic	Latin	Glare, P. G. W. (Ed.). (1982). *Oxford Latin dictionary.* Oxford: Clarendon Press.	D
48	Indo-European: Indo-Iranian	Hindi	Sharma, V. P. (Ed.). (2006). *The Rupa English-Hindi dictionary.* New Delhi: Rupa & Co.	S
49	Indo-European: Indo-Iranian	Balochi	Gilbertson, G. W. (1925). *English Balochi colloquial dictionary.* Hertford: S. Austin & sons.	S

Table S1: (continued)

ID	Grouping	Language	Reference	Form
50	Indo-European: Other Indo-European	Modern Greek	Divry, G. C. (1961). *Modern English-Greek and Greek-English desk dictionary.* New York: D. C. Divry.	D
51	Indo-European: Other Indo-European	Danish	Vinterberg, H. & Axelsen, J. (1965). *McKay's modern Danish-English, English-Danish dictionary.* New York: McKay.	D
56	Indo-Pacific: Central New Guinea	Kâte	Flierl, W. & Strauss, H. (1977). *Kâte dictionary.* Canberra: Australian National University.	D
67	Khoisan	Nama	Haacke, W. H. G. & Eiseb, E. (2002). *A Khoekhoegowab dictionary with an English-Khoekhoegowab index.* Windhoek: Gamsberg Macmillan.	D
68	Na-dene	Chipewyan	Le Goff, L. (1916). *Dictionnaire français-montagnais.* Lyon: Société Saint-Augustin.	D
73	Niger-Kordofanian: Niger Congo: Gur	Mossi	Kinda, J. (2004). *Lexiques spécialisés.* Ouagadougou: Département de linguistique, Université de Ouagadougou.	D
74	Niger-Kordofanian: Niger Congo: Kwa	Yoruba	Wakeman, C. W. (1913). *Dictionary of the Yoruba language.* Lagos: Church Missionary Society Bookshop.	D
76	Nilo-Saharan: Chari-Nile	Dholuo	Odaga, A. (1997). *English-Dholuo dictionary.* Kisumu, Kenya: Lake Publishers & Enterprises.	D
78	Nilo-Saharan: Other Nilo-Saharan	Chikaranga	Louw, C. S. (1915). *A manual of the Chikaranga language.* Bulawayo: Philpott & Collins.	S
79	Oto-Manguean	Popolocan	Krumholz, J. A., Dolson, M. K., & Hernández Ayuso, M. (1995). *Diccionario popoloca de San Juan Atzingo Puebla.* Tucson: Instituto Lingüístico de Verano.	S
80	Penutian: Mayan	Tzotzil	Laughlin, R. M. & Haviland, J. B. (1988). *The great Tzotzil dictionary of Santo Domingo Zinacantán.* Washington, DC: Smithsonian Institution Press.	D
81	Penutian: Other Penutian	Zuni	Newman, S. S. (1958). *Zuni dictionary.* Bloomington: Indiana University.	D
82	Penutian: Other Penutian	Maidu	Shipley, W. F. (1963). *Maidu texts and dictionary.* Berkeley and Los Angeles: University of California Press.	D

Table S1: (continued)

ID	Grouping	Language	Reference	Form
83	Salish	Shuswap	Kuipers, A. H. (1974). *The Shuswap language: Grammar, texts, dictionary.* The Hague: Mouton.	D
84	Sino-Tibetan: Kam-Tai	Bouyei	Esquirol, J. & Williatte, G. (1908). *Essai de dictionnaire dioi-français.* Hong Kong: Imprimerie de la Société des Missions Étrangères.	D
85	Sino-Tibetan: Tibeto-Burman	Newari	Tuladhar, K. (1998). *English-Nepalbhasa wordbook.* Kathmandu: Bhulukha Publications.	D
86	Sino-Tibetan: Tibeto-Burman	Manipuri	Shitaljit, R. K. (1976). *Friends' English to Manipuri dictionary.* Imphal: K. Tombi Singh.	D
87	Sino-Tibetan: Tibeto-Burman	Lahu	Matisoff, J. A. (2006). *English-Lahu lexicon.* Berkeley: University of California Press.	D
88	Sino-Tibetan: Tibeto-Burman	Maru	Clerk, F. V. (1911). *A manual of the Lawngwaw or Maru language.* Rangoon: American Baptist Mission Press.	S
89	Sino-Tibetan: Other Sino-Tibetan	Cantonese	Huang, P. P. (1970). *Cantonese Dictionary.* New Haven: Yale University Press.	S
90	Macro-Siouan	Dakota	Williamson, J. P. (1902). *An English-Dakota dictionary.* New York: American Tract Society.	D
91	Ural-Altaic: Uralic	Hungarian	Országh, L. (1960). *English-Hungarian dictionary.* Budapest, Akadémiai Kiadó.	D
92	Ural-Altaic: Altaic: Turkic	Karagas	Castrén, M. A. (1857). *Versuch einer Koibalischen und Karagassischen Sprachlehre.* St. Petersburg: Buchdruckerei der Kaiserlichen Akademie der Wissenschaften.	D
93	Ural-Altaic: Altaic: Other Altaic	Udihe	Nikolaeva, I. & Tolskaya, M. (2001). *A grammar of Udihe.* Berlin: Mouton de Gruyter.	D
94	Creoles	Pijin	Jourdan, C. (2002). *Pijin: A trilingual cultural dictionary.* Canberra: Australian National University.	D
